# Assay for Glycosaminoglycans by Tandem Mass Spectrometry and its Applications

**DOI:** 10.4172/2155-9872.S2-006

**Published:** 2014-03-01

**Authors:** Shunji Tomatsu, Tsutomu Shimada, Robert W Mason, Joan Kelly, William A LaMarr, Eriko Yasuda, Yuniko Shibata, Hideyuki Futatsumori, Adriana M Montaño, Seiji Yamaguchi, Yasuyuki Suzuki, Tadao Orii

**Affiliations:** 1 Nemours/Alfred I. duPont Hospital for Children, Wilmington, DE, USA; 2 Agilent Technologies, Inc., Wakefield, MA, USA; 3 Central Research Lab., R&D Div. Seikagaku Co. Tokyo, Japan; 4 Department of Pediatrics, Saint Louis University, St. Louis, Missouri, USA; 5 Department of Pediatrics, Shimane University, Izumo, Japan; 6 Medical Education Development Center, Gifu University, Japan; 7 Department of Pediatrics, Gifu University, Gifu, Japan

**Keywords:** Tandem mass spectrometry, Glycosaminoglycans, Mucopolysaccharidoses, Chromatogram, Newborn screening

## Abstract

Glycosaminoglycans (GAGs) are distributed in the whole body and play a variety of important physiological roles associated with inflammation, growth, coagulation, fibrinolysis, lipolysis, and cell-matrix biology. Accumulation of undegraded GAGs in lysosomes gives rise to a distinct clinical syndrome, mucopolysaccharidoses. Measurement of each specific GAG in a variety of specimens is urgently required to understand GAG interaction with other molecules, physiological status of patients, and prognosis and pathogenesis of the disease.

We established a highly sensitive and accurate tandem mass spectrometry (LC-MS/MS) method for measurements of disaccharides derived from four specific GAGs [dermatan sulfate (DS), heparan sulfate (HS), keratan sulfate (KS), and chondroitin sulfate (CS)]. Disaccharides were produced by specific enzyme digestion of each GAG, and quantified by negative ion mode of multiple reaction monitoring. Subclasses of HS and GAGs with identical molecular weights can be separated using a Hypercarbcolumn (2.0 mm×50 mm, 5 μm) with an aectonitrile gradient in ammonium acetate (pH 11.0).

We also developed a GAG assay by RapidFire with tandem mass spectrometry (RF-MS/MS). The RF system consists of an integrated solid phase extraction robot that binds and de-salts samples from assay plates and directly injects them into a MS/MS detector, reducing sample processing time to ten seconds. RF-MS/MS consequently yields much faster throughput than conventional LC-MS/MS-based methods.

However, the RF system does not have a chromatographic step, and therefore, cannot distinguish GAGs that have identical molecular weights. Both methods can be applied to analysis of dried blood spots, blood, and urine specimens.

In this article, we compare the assay methods for GAGs and describe their potential applications.

## Introduction

Glycosaminoglycans (GAGs) comprise chondroitin sulfate (CS), dermatan sulfate (DS), heparan sulfate (HS), keratan sulfate (KS), and hyaluronan. These GAGs are found in various tissues and are major components of the extracellular matrix (ECM). GAGs (except for hyaluronan) are sulfated polysaccharides made of repeating disaccharides, consisting of uronic acid (or galactose) and hexosamines. Polymeric GAGs are covalently bound through a linkage region to core proteins to yield proteoglycans (PGs), which have various biological functions. PGs play important roles in control of growth and differentiation. Particular sulfation patterns in the GAG chains allow interactions, normally of ionic nature, with growth factors. The PG protein cores are not just scaffolds for GAGs: they contain domains that have particular biological activities [1]. Many PGs are thus multifunctional molecules that engage in several different specific interactions simultaneously.

Mucopolysaccharidoses (MPS) are a group of lysosomal storage diseases caused by deficiency of the lysosomal enzymes required for degradation of GAGs. There are 11 known enzyme deficiencies, resulting in seven distinct forms of MPS with a collective incidence of approximately 1 in 25,000 live births. GAG accumulation causes a progressive damage of multiple tissues including brain, lung, heart, liver, kidney, joint, and bone. Most clinical signs and symptoms for MPS patients do not appear immediately after birth but onset of clinical signs and symptoms progresses with age. Although the symptoms and severity of MPS vary with each individual patient and its subtype of MPS, the average life span in most patients is one to two decades if untreated.

Currently, enzyme replacement therapy (ERT), hematopoietic stem cell transplantation, substrate reduction therapy and gene therapy are in clinical use or being investigated in clinical trials for patients with some types of MPS. Starting these treatments at birth or at very early stages has the greatest positive impact on the clinical course of the disease [2]. To provide a better efficacy and quality of life of the patients, early diagnosis and early treatment as well as accurate prognosis and monitoring are essential.

Newborn Screening (NBS) is recognized as an essential, preventive public health program for early identification of diseases in newborn that can affect their long-term health. Tandem mass spectrometry (MS/MS) has evolved as a powerful tool to detect small compounds because detection is based on the mass of the parent compound and a specific fragment(s) of the compound. Other sensitive detection methods, such as amperometric electrochemical detection, do not detect specific compounds and consequently quantitation will be compromised if other compounds co-elute with target compound after chromatographic separation. This is a particular concern in our studies of multiple disaccharides that have similar chromatographic and molecular properties. We developed a method that can assay DS, HS, and KS simultaneously in blood and/or urine samples using high performance liquid chromatography tandem mass spectrometry (LCMS/MS) for MPS [3-10]. The LC-MS/MS method could not only show sensitivity and specificity for detecting all subtypes of MPS but is also able to monitor therapeutic efficacy in MPS patients and animal models [2,6,10-12]. We show here that CS can be also measured simultaneously using a modification of this technique.

The main drawback of the LC step is that the process is time consuming, limiting its utility for the screening of large numbers of NBS samples. The use of the RapidFire (RF) high-throughput mass spectrometry system removes the bottleneck of throughput, while maintaining the quality and reliability of standard MS/MS read-outs. Samples are absorbed to a matrix to concentrate and desalt, and then eluted directly into the MS/MS without chromatographic separation. Each sample is processed in less than ten seconds, yielding much faster throughput than conventional LC-MS/MS based methods. A single 384 well plate can be read in ~45 min, indicating that this RF-MS/MS system can analyze over one million samples annually. Moreover, the RF-MS/MS has been shown to provide sensitivity and specificity equivalent to a scintillation proximity assay [13], and LC-MS/MS system in some applications. [14]. The speed and efficiency of the RF can allow for experiments that would otherwise be deemed “untenable” under normal circumstances. The RF system has been validated as suitable for many drug discoveries [13,15-21], and ADME (Absorption, Distribution, Metabolism and Excretion) based applications [14].

Overall, establishment of GAG assays by tandem mass spectrometry are urgently required to apply to not only clinical indications but basic research. In this article, we describe GAGs assay methods by using LCMS/MS and RF-MS/MS, indicating their application in clinical and basic research.

## Procedures of Tandem Mass Spectrometry for Assay of GAGs

### Standards and enzymes

To digest “polymer” CS, DS, HS, and KS to disaccharides, the enzymes of chondroitinase C, chondroitinase B, heparitinase, and keratanase II, respectively, were provided from Sigma-Aldrich (St. Louis, MO) and Seikagaku Co. (Tokyo, Japan). Chondrosine (Seikagaku Co.) was used as an internal standard (IS), while unsaturated disaccharides, [ΔDiHS-0S, 2-acetamido-2-deoxy-4-O-(4-deoxy-a-L-threo-hex-4-enopyranosyluronic acid)-D-glucose; ΔDiHS-NS, 2-deoxy-2-sulfamino-4-O-(4-deoxy-a-L-threo-hex-4-enopyranosyluronic acid)-D-glucose; ΔDi-4S/6S (DS or CS), 2-acetamido-2-deoxy-4-O-(4-deoxy-a-L-threo-hex-4-enopyranosyluronic acid)-4 or 6-O-sulfo-D-glucose] were used for making standard curves. Stock solutions of ΔDi-6S (250 μg/ml), ΔDi-4S (250 μg/ml), ΔDiHS-0S (100 μg/ml), ΔDiHS-NS (100 μg/ml), ΔDiHS-6S (100 μg/ml), and IS (5 mg/ml) were prepared separately in ddH_2_O. Mono-sulfated KS [KS1; Galβ1-4GlcNAc(6S)] and di-sulfated KS [KS2; Gal(6S)β1-4GlcNAc(6S)] were made by digestion of bovine cornea KS with keratanase II, since these disaccharides were not available. Sixty percent of KS-I was digested with keratanase II and that the ratio of Gal(6S)β1-4GlcNAc(6S) (KS1) toGalβ1-4GlcNAc(6S)(KS2) was 42:58 [22]. A stock solution of digested KS (1000 μg/ml KS1+KS2) in ddH_2_O was prepared.

For standard curves, dilutions of the stock solutions were prepared as follows: ΔDi-6S, ΔDi-4S, ΔDiHS-0S, ΔDiHS-NS, and ΔDiHS-6S (1, 5, 10, 50, 100, 500, and 1000 ng/ml), and digested KS (0.01, 0.05, 0.1, 0.5, 1, 5, and 10 μg/ml), each containing IS solution (50 ng/ml).

**Figure 1:**
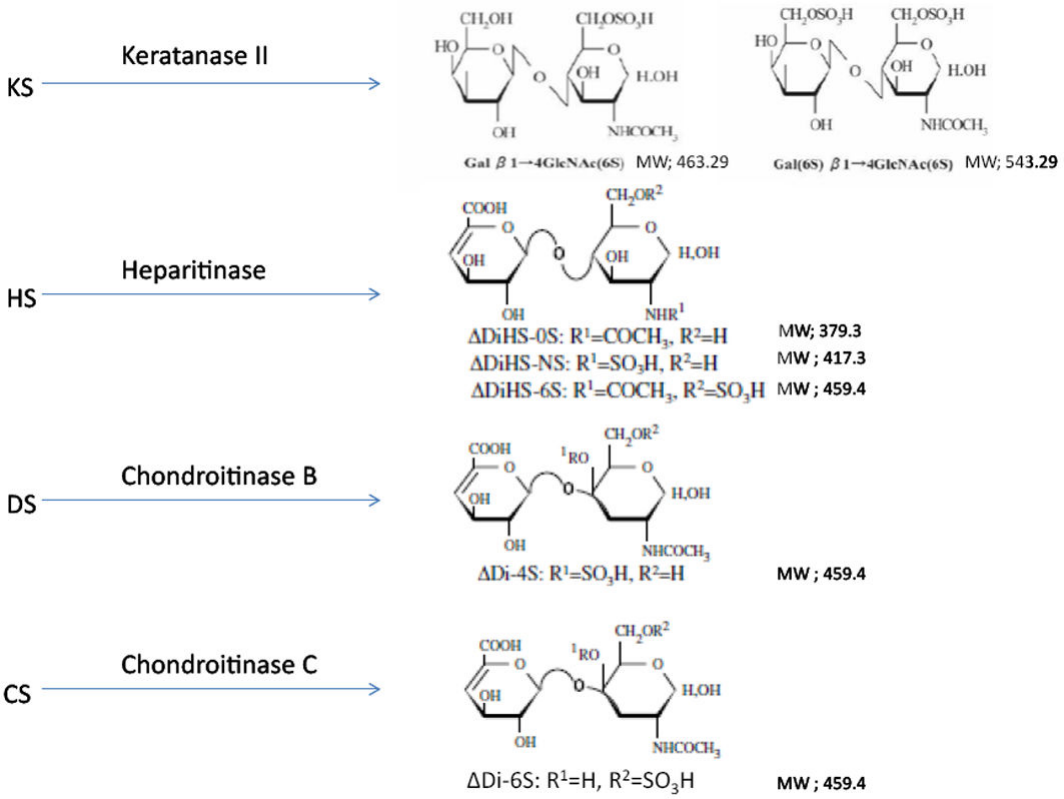
Disaccharides after digestion of GAGs by enzymes. KS: Keratan sulfate; HS: heparan sulfate; DS:dermatan sulfate; CS: chondroitin sulfate.

ΔDi-4S is composed of DS, and ΔDi-C6S derived from CS, while ΔDiHS-0S, ΔDiHS-NS, and ΔDiHS-6S are subclasses of HS.KS comprises KS1 and KS2 (Figure 1). Note that ΔDi-4S, ΔDiHS-6S, and ΔDi-C6S have identical molecular weights.

### Sample preparation

**Figure 2:**
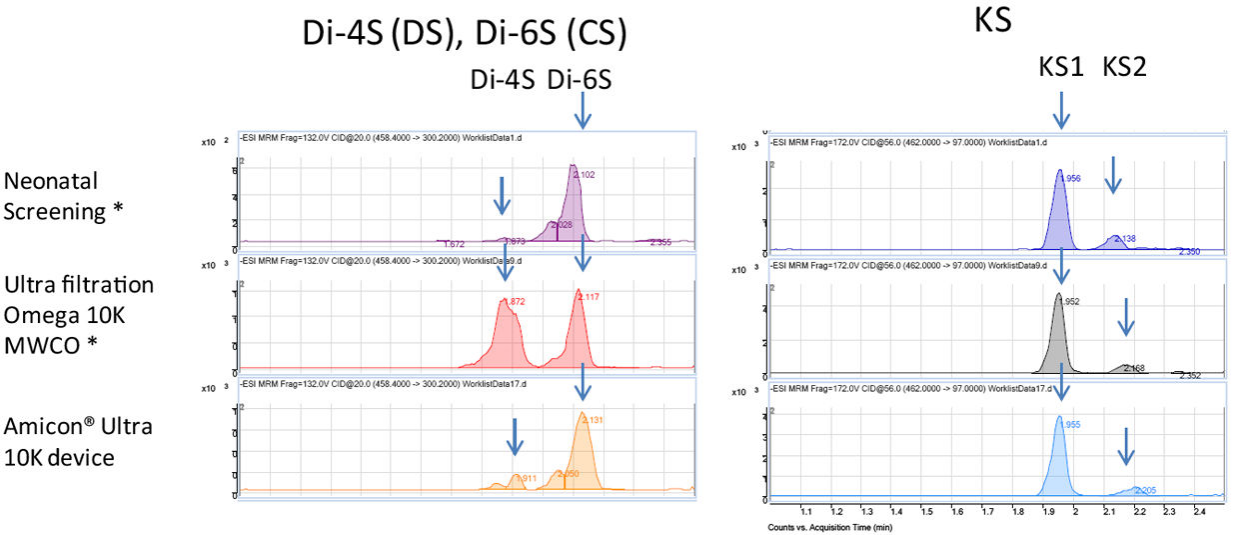
Comparison of disaccharide elution between filters The peak corresponding to Di-4S (DS) in the chromatogram after use of the Ultra filtration Omega 10K filter was much larger than seen for the other filters. The amount of KS1 (mono-sulfated KS) and KS2 (di-sulfated KS) (right panel) was also higher from this filter than the neonatal screening filter (note scale of y-axis). Elution of HS was similar for all 3 filters (data not shown).*AcroPrep™ Advance 96-Well Filter Plates.

Purified polymer CS (shark cartilage), DS (pig skin), HS (bovine kidney), and KS (bovine cornea) were digested by a mixture of chondroitinase C (Sigma-Aldrich Co, St. Louis MO), chondroitinase B, heparitinase, and keratanase II (all other reagents except chondroitinase C provided by Seikagaku Co). Samples to be measured were centrifuged to remove insoluble material. Ten μL of the supernatant was mixed with 90 μL of 50 mM Tris-HCl Buffer (pH 7.0), and then added to a filter well. This was centrifuged at 2,500 g for 15 min to deplete low molecular compounds that could interfere with the assay. The filters were transferred to fresh plates and 20 μL of internal standard (500 ng/mL chondrosine), 20 μL of 50 mM Tris-HCl buffer and 10 μL of chondroitinase C, chondroitinase B, heparitinase, and keratanase II, respectively, (each 2 mU/10 μL of 50 mM Tris-HCl buffer) were added onto each filter. Samples were incubated with shaking at 37°C for 15 hr to digest the GAGs. Filter plates were then centrifuged at 2,500 g for 15 min. Twenty μL of ddH_2_O was added to each flow-through sample, and mixed by vortexing for 10 sec. Samples were stored at -20°C until injection to LC-MS/MS or RF-MS/MS. In a preliminary study we tested three different filter systems for efficiency of extraction and recovery: AcroPrep™ Advance 96-Well Filter Plates, with Ultra filtration Omega 10K(PALL Co., NY, USA); Neonatal Screening, AcroPrept™ Advance 96-Well Filter Plates (PALL Co.); and Amicon Ultra 10K device (Milipore Co.) and monitored recovery of Di4S and KS (Figure 2). The optimized Di-4S assay also detects Di-6S because these disaccharides have the same molecular mass and yield similar fragments. The KS assay detects both mono and disulfated KS because the fragmentor can remove a sulfate from the disulfated form before it enters the mass spectrometer. The filter with the ultrafiltration Omega 10K filters gave the highest yield of disaccharides, especially for Di-4S.

### Equipment

#### Liquid chromatography tandem mass spectrometry (LC-MS/MS)

**Figure 3:**
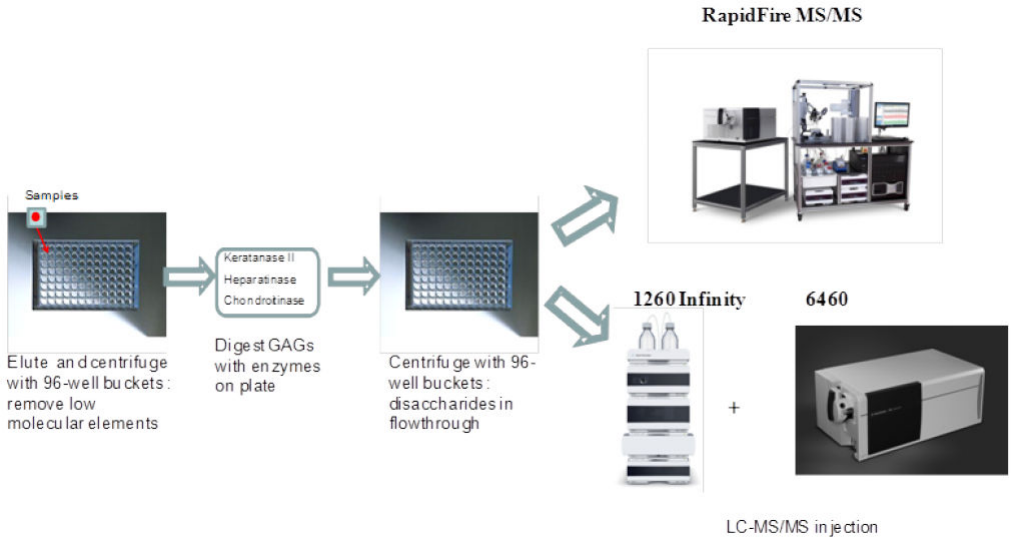
Procedures of LC-MS/MS and RF-MS/MS Before application to LC-MS/MS or RF-MS/MS, all the procedures were performed in the same manner.

The instrument used for high performance liquid chromatography tandem mass spectrometry (LC-MS/MS) was a 1260 infinity LC/6460 Triple Quad (Agilent Technologies, Palo Alto, CA, USA). Samples were removed from the -20°C and were allowed to thaw at an ambient temperature. Sample tubes were quickly spun by using a VWR Galaxy Ministar centrifuge to collect all samples to the bottom of the tube. Twenty-five μL of each sample was transferred to a 96 well polypropylene plate, and diluted with 25 μL of 50 mMTris buffer (pH 7.5). The plate was centrifuged at 3,500 g for 5 min prior to analysis with the 1260 infinity LC /6460 Triple Quad (Figure 3).

#### RapidFire tandem mass spectrometry (RF-MS/MS)

The samples purified by the filters as described above for LC-MS/MS were also analyzed using the RapidFire high-throughput mass spectrometry platform (Agilent Technologies, Inc: Santa Clara, CA). Individual AUC (areas under the curve) for each analyte in each sample was determined using the RapidFire Integrator software. Each sample was prepared and analyzed in duplicate on separate days. All GAG levels were measured and averaged.

### LC-MS/MS: Determination of levels of disaccharides derived from GAGs

#### Chromatography and selectivity

**Figure 4:**
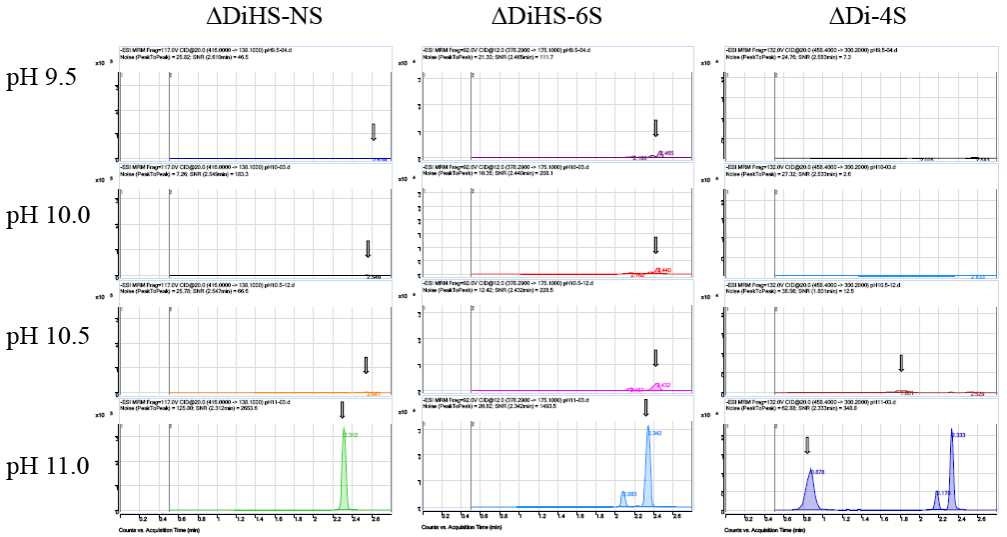
Chromatograms in different pHs for Hypercarb The samples were analyzed by using Hypercarb (2.0 mm×50 mm, 5 μm; Thermo Electron Corp.) with an acetonitrile gradient of 0% to 100% in 5 mM ammonium acetate. The range of pH tested was between pH 9.5 and pH 11.0. The chromatograms indicate that pH 11.0 provides the highest peaks for ΔDiHS-NS, ΔDiHS-6S, and ΔDi-4S (DS).

**Figure 5:**
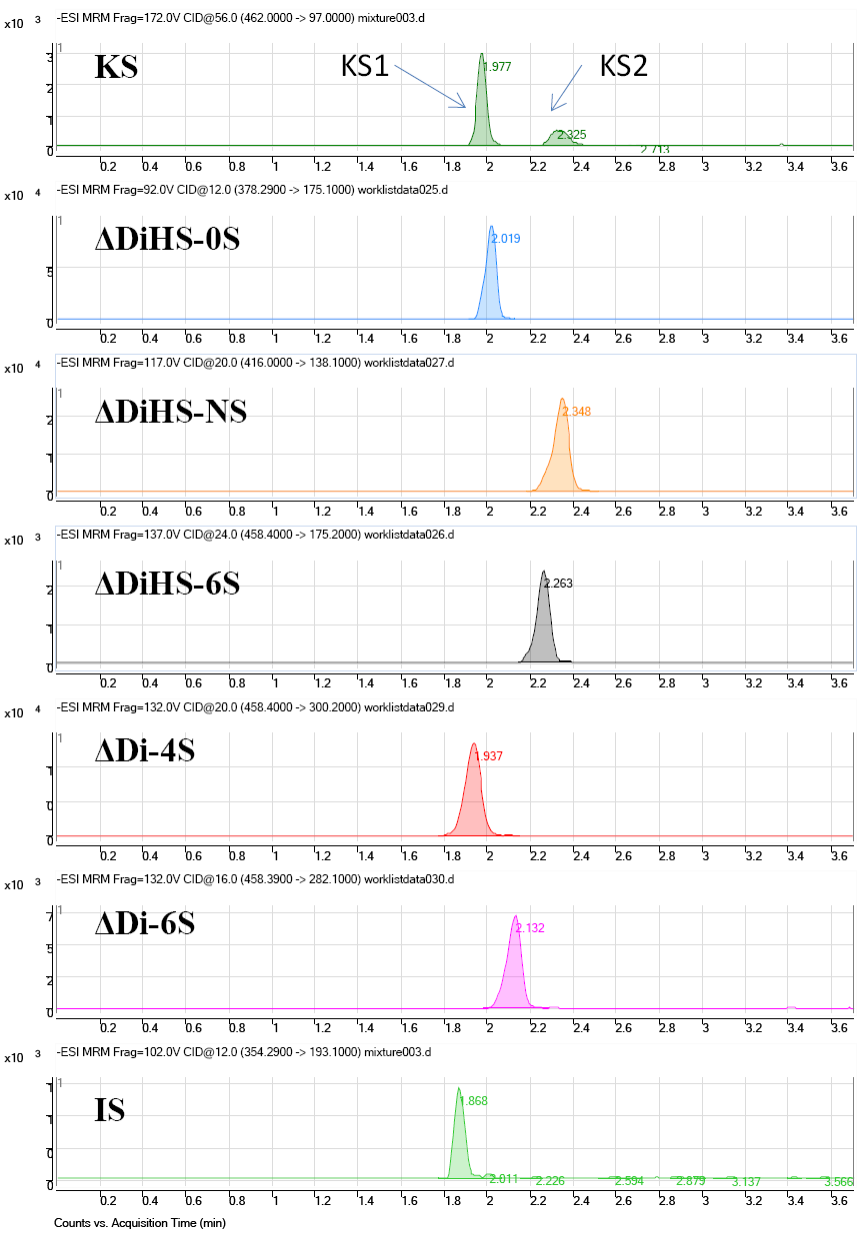
Chromatograms of disaccharides Chromatograms for disaccharides of KS 1 and 2 (digested bovine cornea), ΔDiHS-NS, ΔDiHS-0S, ΔDiHS-6S, ΔDi-4S (DS), ΔDi-6S (C6S), and chondrosine (IS). Polymer KS was separated with mono-sulfated KS [KS1: Galβ1-4GlcNAc(6S)] and di-sulfated KS [KS2: Gal(6S)β1- 4GlcNAc(6S)] after digestion by keratanase II. Equipment: 6460 Triple Quad MS/MS with 1260 infinity LC (Agilent Technologies). IS: Internal Standard.

Disaccharides were separated on a Hypercarb (2.0 mm×50 mm, 5 μm; Thermo Electron Corp.) column and eluted with an acetonitrile gradient of 0% to 100% in 5 mM ammonium acetate. Signals for ΔDiHS-6S, ΔDi-4S (DS), and ΔDi-6S (C6S) were very low at pH values below 11 (Figure 4), probably due to poor ionization of the sugars. Using purified preparations ofΔDiHS-0S, ΔDiHS-NS, ΔDiHS-6S, ΔDi-4S, and ΔDi-6S, we optimized MRMs for each individual sugar and obtained single peaks corresponding to the pure compound (Figure 5). All disaccharides are eluted in less than 3 min (Figure 5). As discussed earlier, although ΔDiHS-6S, ΔDi-4S (DS), and ΔDi-6S (C6S) have the same molecular weights, they elute at different times from the column, so they can be quantified separately. Furthermore, the elution position of Di-4S varies dependent upon the sample preparation, and elutes at 0.9 min after extraction from some samples (e.g. Figure 4). All other disaccharides consistently eluted as seen for the standards. As discussed earlier, the detection method for mono-sulfated Galβ1-4GlcNAc(6S) (KS1) also detected some Gal(6S) β1-4GlcNAc(6S) (KS2), due to loss of a sulfate, but the two forms were clearly separated by chromatography.

#### Calibration curves

**Figure 6:**
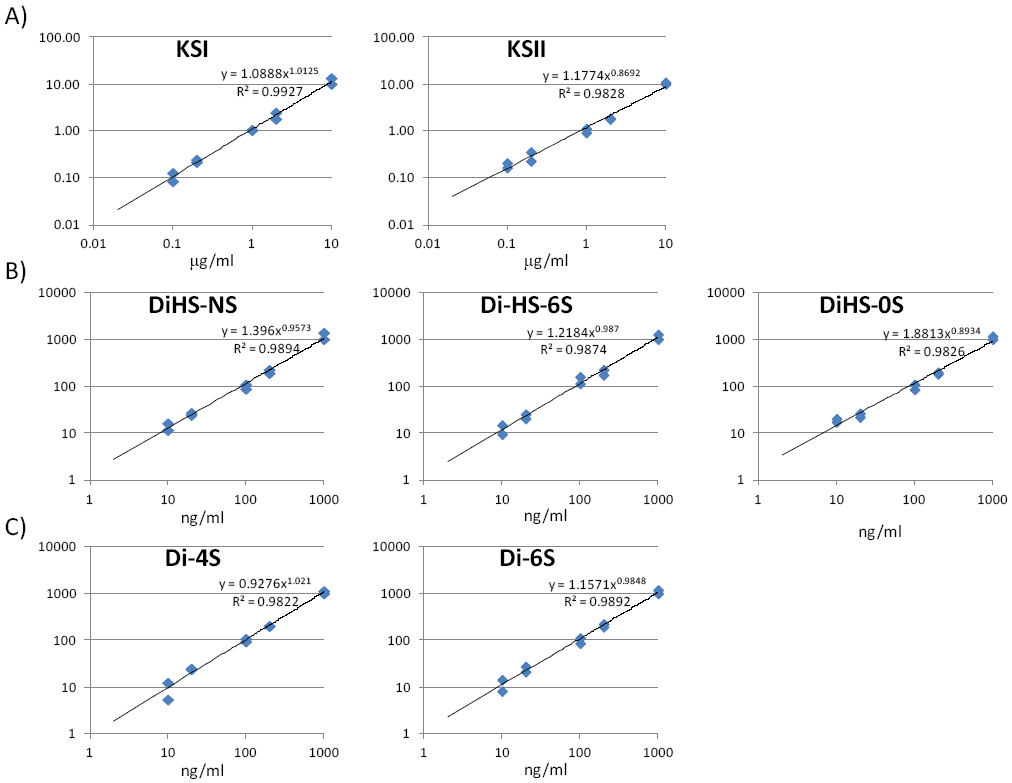
Calibration curves for each disaccharide by LC-MS/MS X-axis shows actual concentration of disaccharide, while Y-axis shows calculated concentration. A) Galβ1-4GlcNAc(6S); KS1, Gal(6S)β1-4GlcNAc(6S); KS2, B) ΔDiHS-0S, ΔDiHS-NS, and ΔDiHS-6S, C) ΔDi-6S; C6S and ΔDi-4S; DS.

Calibration parameters of disaccharides derived from CS, DS, HS, and KS were assessed. Calibration curves for Galβ1-4GlcNAc(6S) (KS1), Gal(6S)β1-4GlcNAc(6S) (KS2), ΔDiHS-0S (H0S), ΔDiHS-NS (HNS), and ΔDiHS-6S (H6S), ΔDi-6S (C6S), and ΔDi-4S (DS) are linear over the concentration ranges of 0.1to 10 μg/ml, 0.1 to 10 μg/ml, 10 to 1,000 ng/ml, 10 to 1,000 ng/ml, 10 to 1,000 ng/ml, 10 to 1,000 ng/ml, and 10 to 1,000 ng/ml, respectively. The correlation coefficients of determination (r) were not less than 0.98 (Figure 6).

#### Precision

The results of intra- and inter-assay precision for ΔDiHS-0S, ΔDiHS-NS, and ΔDiHS-6S, ΔDi-6S, and ΔDi-4S in control serum are as follows. The intra-assay precision values/coefficient of variation (CV) determined from analysis of ΔDiHS-0S, ΔDiHS-NS, ΔDiHS-6S, ΔDi-6S, and ΔDi-4S for control serum are less than 8.9, 1.7, 11.7, 11.9, and 12.1%, respectively. The inter-assay precision values/CVs for these disaccharides in control serum are less than 7.0, 0.6, 12.2, 12.2, and 12.4%, respectively. The intra-assay precision values/CVs for Galβ1-4GlcNAc(6S) and Gal(6S)β1-4GlcNAc(6S) in control serum were less than 6.8 and 14.6% andinter-assay precision values/CVs were less than 6.5 and 14.3% (Table 1). These results demonstrate the reproducibility and accuracy of the method.

### RF-MS/MS: Disaccharide determination derived from GAGs

#### Chromatography

**Figure 7:**
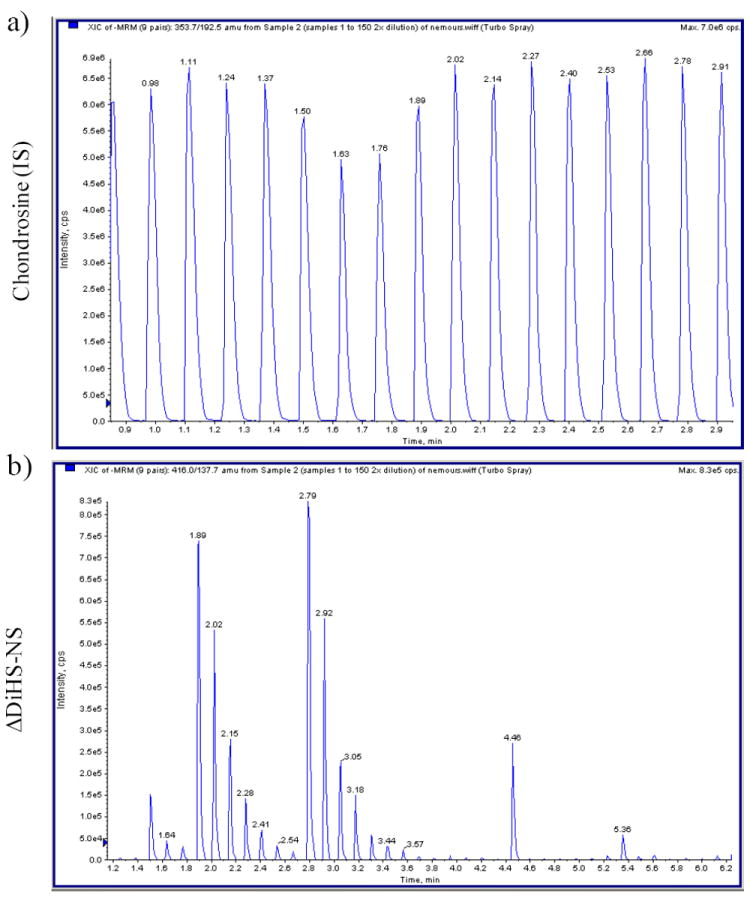
Chromatograms of RF-MS/MS a) Multiple injections of chondosine (Q1; 353.7, Q3; 192.5) with the same concentration shows 8 peaks per min. b) Multiple injections of ΔDiHS-NS (Q1; 416.0, Q3; 137.7) with a series of dilutions in duplicate shows seven gradient peaks per a set of dilutions (as well as other surrounding samples).

The RF system does not have a chromatographic separation step but rather samples are first bound to solid phase matrix for desalting and concentration and then eluted directly into an MS/MS system. Consequently, ΔDiHS-6S, ΔDi-4S, and ΔDi-6S cannot be distingished because they have identical molecular weights and give similar mrms. However the MS/MS can distinguish and quantify combinations of different disaccharides (for example see chondrosine and ΔDiHS-NS in Figure 7).

#### Calibration curves

**Figure 8:**
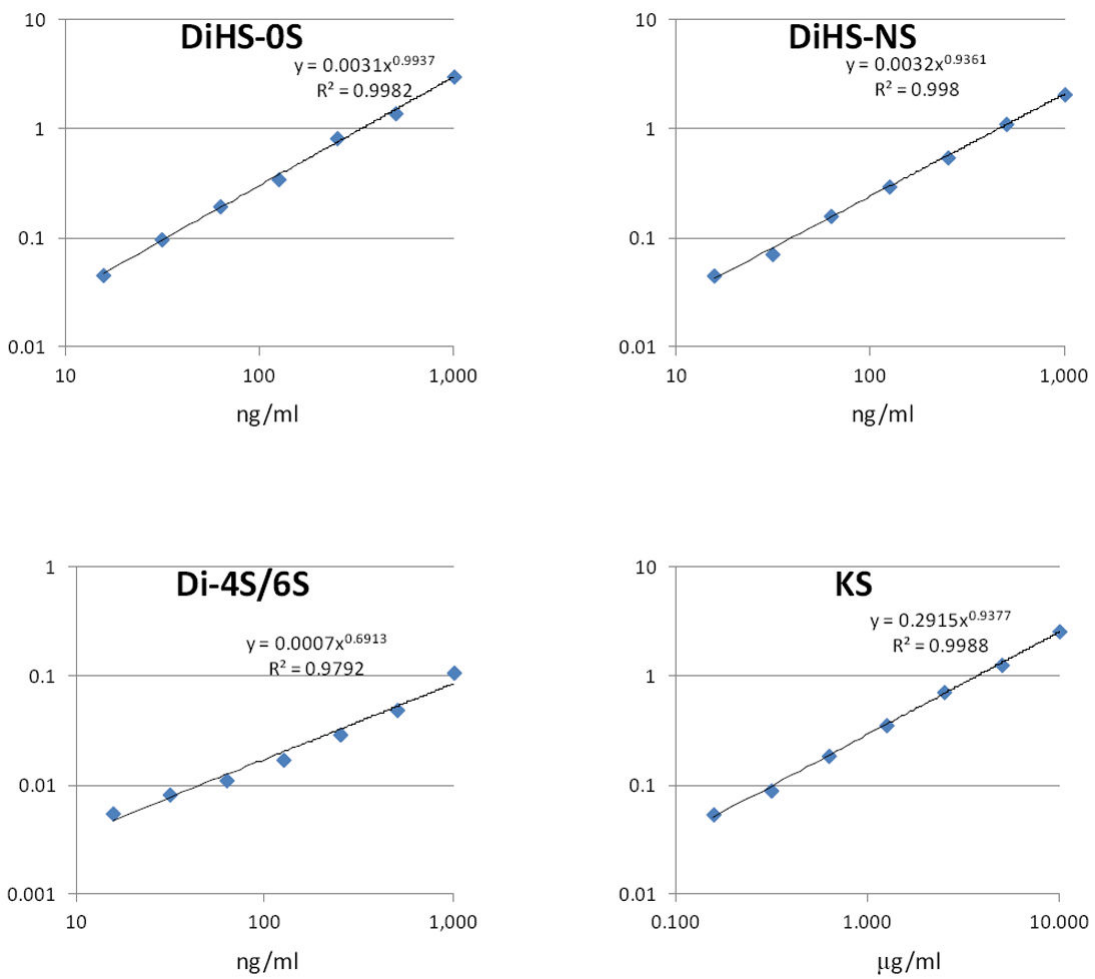
Calibration curves for each disaccharide by RF-MS/MS. X-axis shows actual concentration of disaccharide in solution (ng/ml), while Y-axis shows measured AUC (arbitrary units). KS comprises KS1 and KS2, and ΔDi-4S/6S comprises DS and DiHS-6S.

Calibration parameters of disaccharides derived from DS, HS, and KS were assessed. Calibration curves for KS, ΔDiHS-0S, ΔDiHS-NS, and ΔDi-4S/6S are linear over the concentration ranges of 0.156 to 10.0 μg/ml, 16 to 1,000 ng/ml, 16 to 1,000 ng/ml, and 16 to 1,000 ng/ml, respectively. The correlation coefficients of determination (r) are 0.98 or more (Figure 8).

#### Precision

The results of intra- and inter-assay precision for ΔDiHS-0S, ΔDiHS-NS, and ΔDi-4S/6S in control serum are as follows. The disaccharide (ΔDi-4S) is detected by the same ions (m/z 462/97) as those of ΔDiHS-6S. Thus, the total concentration of disaccharides expressed as ΔDi-4S includes both ΔDi-4S and ΔDiHS-6S. The intraassay precision values/coefficient of variation (CV) determined from analysis of ΔDiHS-0S, ΔDiHS-NS, and ΔDi-4S for control serum are less than 6.7, 7.9, and 15.8%, respectively. The inter-assay precision values/CVs for these disaccharides in control serum are less than 5.2, 6.8, and 14.8%, respectively (Table 2). The intra-assay precision values/CVs for Galβ1-4GlcNAc(6S) and Gal(6S)β1-4GlcNAc(6S) in control serum were less than 8.2 and 5.3% andinter-assay precision values/CVs were less than 6.6 and 5.7%. These results demonstrate the reproducibility and accuracy of the method.

#### Correlation of GAG levels between LC-MS/MS and RF-MS/MS

**Figure 9:**
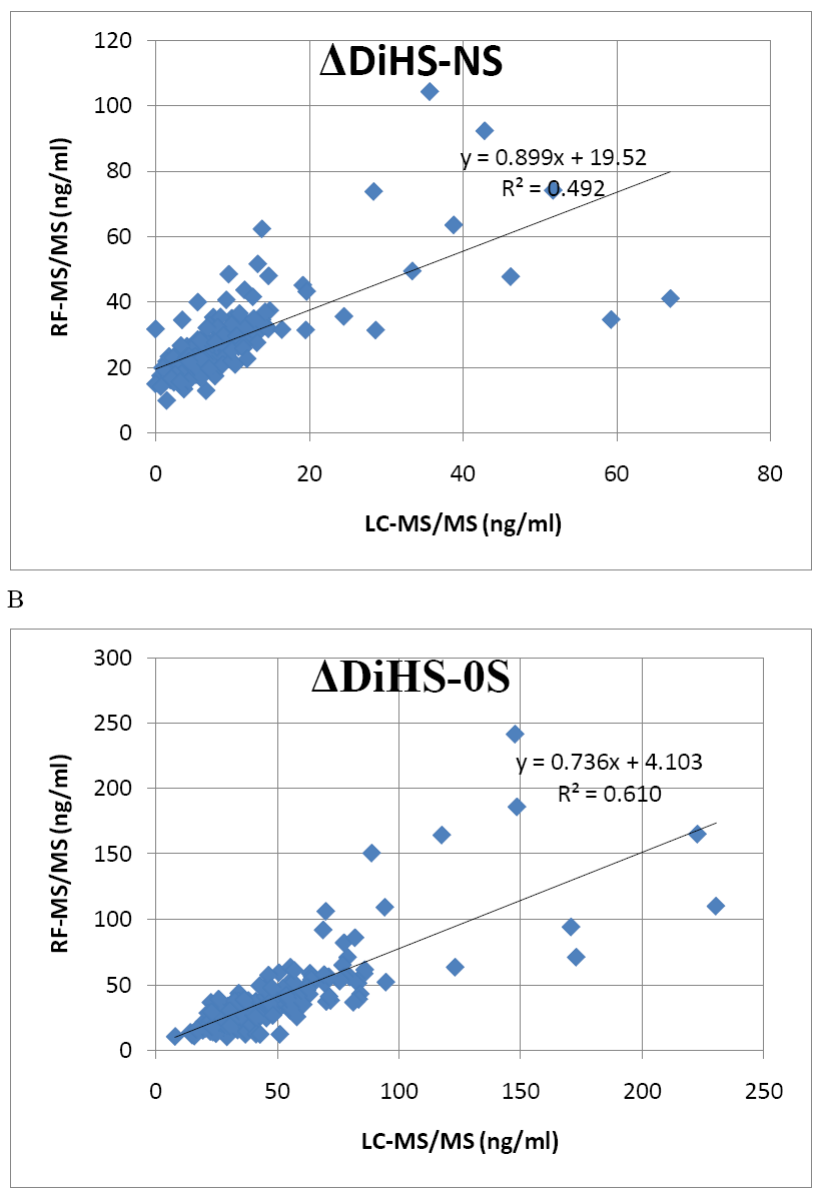
Correlation in GAG concentration between LC-MS/MS and RF-MS/MS A. ΔDiHS-NS concentration. Correlation in blood (plasma or serum) ΔDiHS-NS of human subjects between LC-MS/MS and RF-MS/MS is significant (n=187, p<0.0001). B. ΔDiHS-0S concentration. Correlation in blood (plasma or serum) ΔDiHS-0S of human subjects between LC-MS/MS and RF-MS/MS is significant (n=187, p<0.0001).

We analyzed 59 different plasma samples for DiHS-NS, DiHS-0S, Di-4S, and KS by both LC-MS/MS and RF-MS/MS. The correlation between LC-MS/MS and RF-MS/MS determinations was tested by a simple linear regression analysis. We interpreted correlation strength as outlined by Johnston [23]. Briefly, correlations are interpreted based on r values as follows: 0.0 to 0.2, very weak to negligible correlation; 0.2 to 0.4, weak correlation; 0.4 to 0.7, moderate correlation; 0.7 to 0.9, strong correlation. Data points that were greater than three standard deviations from the mean for each assay were considered outliers. Analysis was performed using SPSS for Windows (version 17.0, SPSS Inc., Chicago, IL, USA). The moderate correlation in serum ΔDiHS-NS and ΔDiHS-0S measurements of control subjects between LC-MS/MS and RF-MS/MS indicates that results from each assay are comparable (Figure 9). Measurements of KS also showed moderate correlation (data not shown).

## Applications of GAG assays

**Figure 10:**
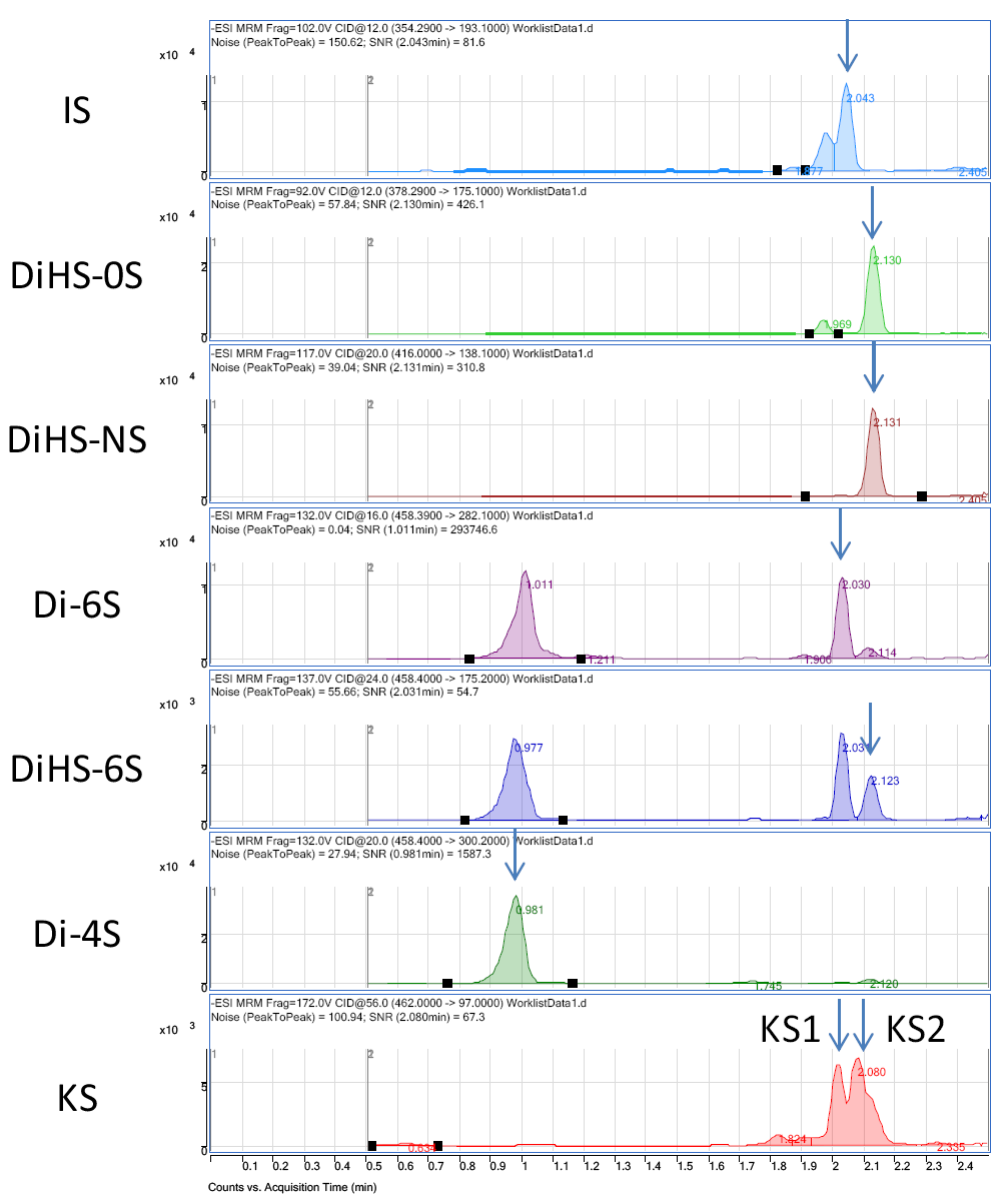
Chromatograms of disaccharides from the rat model with MPS VI (urine) ΔDi-4S comprises DS, and KS comprises KS1 and KS2.

**Figure 11:**
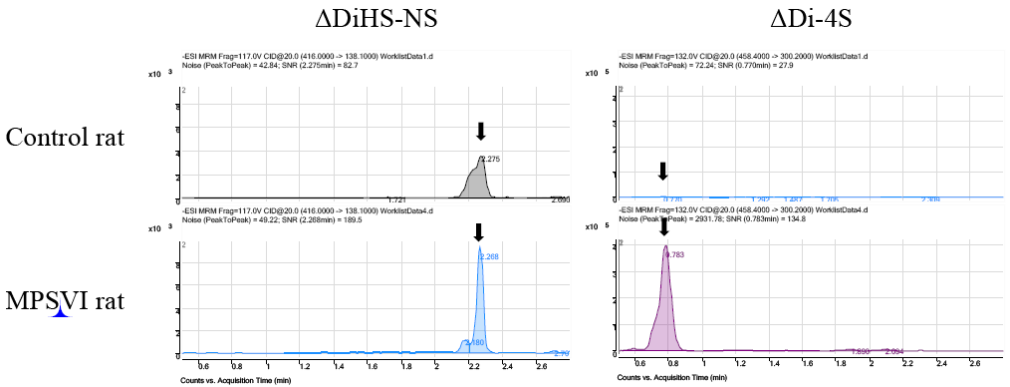
Chromatograms of urine ΔDiHS-NS and ΔDi-4S from the rat with MPS VI and the age-matched control rat ΔDi-4S (DS) in MPS VI rat is markedly elevated, compared with that in age-matched control rat.

Disaccharide GAGs assayed by LC-MS/MS have been used for diagnosis, screening, prognosis of clinical severity, and assessment of therapeutic efficacy in human patients and animal models with MPS [2,9-12,22,24-27]. In Figure 10, peaks corresponding to expected elution of each disaccharide are marked with arrows. MRM Parameters designed to detect Di-6S and DiHS-6S also detect Di-4S whereas the parameters specifically designed to detect Di-4S do not detect the low levels of Di-6S and DiHS-6S in these samples. A clear difference in Di-4S level between a rat with MPS VI and a control rat was observed (Figure 11).

## Discussion

Our studies have demonstrated that an RF-MS/MS platform is capable of detecting disaccharides of ΔDiHS-0S, ΔDiHS-NS, ΔDi-4S/6S, and KS with similar reproducibility, sensitivity, and specificity as LCMS/MS [3-10], suggesting that the LC component may not be essential for detection and identification of these disaccharides. The strong correlation between the two methods for detection of disaccharides extracted from plasma indicates that non-specific interactions or signal quenching are not major concerns for the RF system for this application. The filter system used in this study to remove low molecular weight components of serum and then to separate digested disaccharides from larger molecules appears to eliminate the need for further purification prior to MS/MS analysis. RF-MS/MS provides 25-100 fold faster throughput compared to conventional LC-MS/MS, and consequently may be more suitable for mass screening applications such as NBS.

RF-MS/MS may not be useful for all applications or tissue samples. As there is no separation step, interfering compounds will not be distinguishable from the compound under study. We found that extracts of rat urine contained a number of interfering peaks, including one that had the same MRM as the internal standard (Figure 10). The significance of these false signals to this limited study require further clarification, but the data emphasize the need to ensure that samples will not give excessive levels or numbers of false positive signals prior to establishment of a RF technique.

RF-MS/MS cannot distinguish ΔDi-4S, ΔDi-6S, and ΔDiHS-6S that have the same molecular weight, so LC-MS/MS will be required if quantities of specific disaccharides are required.

GAG(s) play an important physiological role as PG and are associated with other common diseases such as osteoarthritis (OA) [28-31], ligament injury or trauma in the knee [32,33], spinal cord injury [34-36], and diabetes mellitus (DM) [37-39]. GAG assay by tandem mass spectrometry should be applied to diagnosis, prognosis, and therapeutic efficacy of these diseases. The LC-MS/MS method is also needed to investigate sulfation levels of HS and KS that can result in functional alterations of proteoglycans. RF-MS/MS will be more valuable for situations where rapid throughput is necessary such as in newborn screening.

GAGs are long, unbranched polysaccharides, and have a high negative charge because of acidic sugar residues and/or modifications by sulfate groups. The acidic sugar alternates with an amino sugar in repeated disaccharide units. The GAGs adopt an extended conformation, attract cations, and bind water. Hydrated GAG gels enable joints and tissues to absorb large pressure changes.

After synthesis, PGs are transported from the Golgi to their destinations; ECM, the cell surface or intracellular organelles. Such determinant transport requires mechanisms for recognition, sorting and delivery. These mechanisms are critical in cells such as epithelial cells and neurons, where the cell membrane comprises separate domains. Recognition and sorting require determinant factors in the GAG chains and/or in the PG protein cores. Thus, the physiological role of GAGs is dependent upon tissue and/or cell type. Sulfation level of GAG(s) also varies with age, tissue, and pathological status. For instance, KS is almost always sulfated on C (6) of GlcNAc, while C (6) of Gal is sulfated to a variable extent, depending on the tissue and age [40]. KS chains of fibromodulin in articular cartilage are highly sulfated compared to that in cornea [41,42], and the levels of sulfation in cornea and cartilage increase during normal aging [43,44]. The mechanism of alteration of their sulfation levels remains unknown. However, corneas in patients with macular corneal dystrophy (MCD) have been reported to synthesize low-sulfated KS, resulting in corneal clouding, and sulfate groups attached to KS play critical roles in maintaining corneal transparency [45]. Investigation of sulfation level in each disease and physiological status should help elucidate the mechanism of roles of GAGs.

Thus, GAGs assayed by tandem mass spectrometry can be used as biomarkers not only for diagnosis, prognosis, and monitoring therapeutic effects for MPS but may also prove valuable for study of other diseases such as OA, DM, and spinal cord injury and physiological roles. The analysis technique should also be applicable to other species.

The mechanism of alteration of GAGs levels and their sulfation levels remains unknown. Investigation of each GAG level and its sulfation level in each disease and physiological status should help elucidate the mechanism of secondary alteration of GAGs and their roles.

## Conclusion

In conclusion, both LC-MS/MS and RF-MS/MS methods provide a comparable sensitivity and accuracy for simultaneous measurement of three GAGs (DS, HS, and KS). Advantage of use of LC-MS/MS is that ΔDi-6S (C6S), ΔDi-4S (DS), and DiHS-6S with the same molecular weight can be separated leading to accuracy and specificity of the differential diagnosis although it takes 3 min per sample to be analyzed. The RapidFire platform should provide both high throughput and economic advantages as a screening system for MPS, when compared with the conventional LC-MS/MS methodologies and has the potential of being applied to screening for other inherited metabolic disorders, although this system cannot recognize molecules with the same molecular weight separately.

## Figures and Tables

**Table 1:** Intra- and inter-day precision of the method for determination of disaccharides in human control serum (LC-MS/MS).

	Intra-assay precision (n=3) CV%	Inter-assay precision (n=5) CV%
ΔDiHS-OS	8.9	7.0
ΔDiHS-NS	1.7	0.6
DiHS-6S	11.7	12.2
ΔDi-6S	12.1	12.2
ΔDi-4S	11.9	12.4
Galβ1-4GlcNAc(6S)	6.8	6.5
Gal(6S)β1-4GlcNAc(6S)	14.6	14.3

CV=coefficient of variation

**Table 2:** Intra- and inter-day precision of the method for determination of disaccharides in human control serum (RF-MS/MS).

	Intra-assay precision (n=3) CV%	Inter-assay precision (n=5) CV%
ΔDiHS-OS	6.7	5.2
ΔDiHS-NS	7.9	6.8
ΔDi-4S	15.8	14.8
KS	9.6	9.5

CV=coefficient of variation

## Abbreviations

CS: Chondroitin Sulfate
CNS: Central Nervous System
DBS: Dried Blood Spot
DM: Diabetes Mellitus
DMB: Dimethyl Methylene Blue
DS: Dermatan Sulfate
ERT: enzyme replacement therapy
GAG: Glycosaminoglycan
HS: Heparan Sulfate
KS: Keratan Sulfate
KS1: Galβ1-4GlcNAc(6S)
KS2: Gal(6S)β1-4GlcNAc(6S)
LC-MS/MS: High Performance Liquid Chromatography Tandem Mass Spectrometry
MCD: macular corneal dystrophy
MPS: Mucopolysaccharidoses
MRM: Multiple Reaction Monitoring
OA: Osteoarthritis
NBS: Newborn Screening
PG: Proteoglycan
RF-MS/MS: RapidFire Tandem Mass Spectrometry
